# Notices for December

**Published:** 1869-12

**Authors:** 


### NOTICES FOR DECEMBER.

THE American Tract Society, 27 Cornhill, Boston, have issued BIBLE SKETCHES and their Teachings, for young people, by Samuel Green, B. A. Second series. From the Israelites' entrance into Canaan, to the close of the Old Testament. 16mo, 320 pp. An excellent book.

The American Tract Society, New York, have published a delightful book for Children, 198 pp., 16mo, entitled Bertie's Birth-Day Present, or Patience Rewarded; with illustrations. Buy it for the little ones. Also, "Thoughts on Holy Scripture," by Francis Bacon, Lord Chancellor of England. Compiled by Rev. John G. Hall. 12mo, 408 pp., with a copious index- A book for every Christian toread.

Prospectus of the United States and West India Fresh Meat and Fruit Company, organized March 26, 1869. Room 25, Merchants' Exchange, Philadelphia. If this enterprise is wisely conducted, it will be of incalculable advantage, both to the poor and rich, in saving to the world millions of dollars worth of luscious fruits and nourishing meats, and will be the best "stock" ever offered, because there is money in it, as well as health and comfort and abundance.

The Hartford Publishing Company at Hartford, Conn., will soon have ready for agents, to be sold by subscription only, Mrs. Ellett's new book, "The Court Circles of the Republic." It is the social life of our country for fifty years, the social life of distinguished persons at Washington; and, from the index, of contents about prominent persons, it will be sought after by multitudes. It says Douglass was a suitor of Mary Tod, but she became Mrs. Lincoln. Louis Phillippe wooed Mrs. Bingham; had he won her, how different might have been the world's history, &c., &c.

Carbolic Acid, its Action and Uses, by Charles F. J. Lehlbach, M.D., of Newark, N. J.; its value in wounds, burns, scalds, carbuncles, &c.; and although but a pamphlet of 18 pages, ought to be read by every physician in the land, and by all of general intelligence. Twenty-five cents will secure it to be sent free by mail.

Travellers' Official Guide of the Railways of the United States and Canada. 74 Fulton St., New York, compiled and edited by Edward Vernon; contains Time-tables of all Railroads, Ocean and Inland Steamers; maps of lines, offices, and officers, index of towns, villages, &c., and is a most valuable companion for all travellers. 25 cents.

William Wood & Co., 61 Walker St., New York, have issued a number of valuable works on Microscopic Science, which is drawing increased attention and study throughout the civilized world, and is destined to show that what has been hitherto the unseen world is quite as wonderful and as beautiful, exhibits as much the power and wisdom and goodness of God as the worlds which are visible to the naked eye. The same house, "Membrane Tympani," or the Ear in Health and Disease, illustrarted by 24 Chromo Lithographs, with clinical contributions to the diagnosis and treatment of diseases of the ear, with Supplement, by Dr. Adam Politzer of the University of Vienna. Translated by A. Mathewson and H. G. Newton, M.D., assistant surgeons of the Brooklyn Eye and Ear Hospital.

Lindsay & Blakeston have done a great service to the medical profession in publishing "The Book of Prescriptions," containing three thousand prescriptions, collected from the practice of the most eminent physicians and surgeons, English, French and American; with an Index of Diseases and Remedies, by Henry Beeasley, author of the "Druggists' Receipt Book," and of "The Medical Formula." Pp. 562, 8vo.

Lee & Shepard, of Boston, have published "An American Woman in Europe," by Mrs. S. R. Urbino, which contains very much that every traveller will read with instruction and profit, as to places, persons, and prices, &c. Also, Patty Gray's Journey from Boston to Baltimore, by Mrs. Caroline A. Dall; a beautiful story for children.

Aunt Dina's Pledge, by Miss Mary Dwinell Chellis, published by the National Temperance Society, 172 William St., New York, a very amusing and instructive narrative.

"Good Health," $2.00 a year, published by Alexander Moore, Boston, Mass., for November, has many excellent articles in it relative to health and disease. 20 cents a number.

### ONE HUNDRED THOUSAND SUBSCRIBERS.

As we enter our seventeenth year, we hope, from the numbers of our new subscribers coming in daily, that we shall treble our subscription list. The advantages of Clubbing with the First-class Journals, the adition of thrice our usual matter, the Award of Expensive Engravings, and our Burglar Proof Lock, afford subscribers advantages never before offered. Making our Journal for 1870 a Pictorial, illustrating the Sciences and Mechanical Arts, as well as the Health Department, is also a new advance of the times. We have the satisfaction of knowing that though we have not yet made any noted change in our paper, the demand for it through the American and New York News Companies will be greatly increased, and we feel assured that in a short time, from the improved Journal of 1870, our subscribtions will come in encouragingly.

Address *subscription* and all *business letters* to Hall's Journal of Health, 176 Broadway, and *professional letters* to Dr. W. W. Hall, 176 Broadway, New York.

### CLUBS.

Hall's Journal of Health, Art and Science, for 1870, double its former size, with Pictorial Illustrations, clubs with all papers for $1.00 above their subscription. Splendid Engravings are offered to single subscribers. Club subscribers will not be entitled to engravings or other premiums.

### CHANGE !!!

The January number will be increased in size and contain three times the amount of reading matter at the same price as at present; this increase in the amount of reading will be divided among different departments, scientific, agricultural and general literature, the object being to give a wider scope to our Journal and make it more generally useful.

What pertains to the Health Department will be written for by the Editor of the Journal of Health exclusively.

The other departments will be written for by others and selections made from other sources. Over these departments the editor purposes to exercise control, in this one thing, that no line or word or syllable shall be against good morals or against the religious sentiment of others.

In writing for "Hall's Journal of Health" for sixteen years, it is a great comfort to feel that nothing has been said against woman, the clergy, the Sabbath-day, our Holy Religion or the Bible; but on the contrary, these have been sustained, upheld and honored wherever there has been an opportunity. And it is some satisfaction to feel that in the enlargement, occasion will be offered to take a more decided stand in these directions, for it is believed that the time has arrived when it becomes all the good to draw a sharper line of demarcation as to words and conduct and profession. Water and oil can never coalesce, neither can religion and worldliness; and he is the most perfect model of a man, who stands out most distinctly, most unmistakably, as a Bible Christian.

The magazine and the newspaper have become the family teacher, are excluding better and safer reading; and it becomes every parent who has a regard for the religious welfare of his children, to see to it, that the publications which are placed on the parlor table, CAN BE TRUSTED as never having by any possibility, any word prejudicial to the religion of the Bible; many there are which avoid these things in the main; but every now and then give a secret stab, or a malignant side thrust, all the more effective for evil, by the measure of influence gained over the reader by reason of the general better tenor of the teachings. Graham's Magazine now discontinued, Arthur's and Godey's are all safe monthlies in the direction named, and some others. We certainly know that some of the most pretensious, prove by their occasional utterances, that at heart, their conductors are enemies of the Christian religion. It will be our earnest and steady endeavor, that such a charge shall never be truthfully laid at our door.

Subscriptions to our Journal end with the December number. Those who wish to subscribe for 1870 are requested to send their names without delay with a Post Office order, otherwise at their own risk. The January number will be issued during the first week of December; $1.50 a year. Any back numbers not received will be cheerfully supplied without charge, or sent post paid for fifteen cents. Bound vols. for 1869 or any past year, sent post paid for $1.75. Address only professional letters to Dr. W. W. Hall; all others, for subscriptions, books, &c., to Hall's Journal of Health, 176 Broadway, New York.

WM. WOOD & CO. have published "ANAL FISSURE, its Aetiology, Pathology, Diagnosis and Treatment," by William Bodenhamer, A.M. M.D., author of Congenital Diseases of the Rectum, and Professor of Diseases of the Rectum and Contiguous Textures, 199 pp. 8vo., illustrated by numerous cases and drawings. The editor, after a personal acquaintance with the Author of more than thirty years, and a familiarity with his mode of practice, does not hesitate to pronounce Dr. Bodenhamer as the most successful living practitioner at home or abroad in ANAL FISSURE, FISTULA, STRICTURE of the bowels, PILES.

His manipulations performed in a manner peculiar to himself, are shown and communicated to the Profession with great courtesy and cheerfulness when called upon at his residence, 237 5th Ave., New York; he has not only obtained its confidence and respect here, but has drawn to himself an amount of practice from a list of patients of which he may well be proud, members of various state legislatures, governors, senators, officers in the Army and Navy, and from all the professions, and we feel that we are doing our readers a very great service in making these statements, (without the author's knowledge) in case any of them should need his skill in the direction referred to.

As the cases given are very instructive to the general reader, and the causes of these most distressing diseases are plainly stated, and by knowing them the maladies may be avoided, we will keep the book for sale in our office, or will send it post-paid on having $2.25 remitted in a post office order, for safety, to address of HALL'S JOURNAL OF HEALTH, 176 Broadway, New York.

